# Analysis of Newspaper Coverage of Active Aging through the Lens of the 2002 World Health Organization Active Ageing Report: A Policy Framework and the 2010 Toronto Charter for Physical Activity: A Global Call for Action

**DOI:** 10.3390/ijerph10126799

**Published:** 2013-12-05

**Authors:** Boushra Abdullah, Gregor Wolbring

**Affiliations:** 1 Faculty of Medicine, University of Calgary, 3330 Hospital Drive NW, Calgary AB T2N 4N1, Canada; E-Mail: bmabdull@ucalgary.ca; 2 Faculty of Medicine, Department of Community Health Sciences, Stream of Community Rehabilitation and Disability Studies, University of Calgary, 3330 Hospital Drive NW, Calgary AB T2N 4N1, Canada

**Keywords:** active aging, aging well, healthy aging, natural aging, successful aging, *Toronto Charter for Physical Activity: A Global Call for Actio*n, World Health Organization, *Active Ageing: A Policy Framework*

## Abstract

As populations continue to grow older, efforts to support the process of aging well are important goals. Various synonyms are used to cover aging well, such as active aging. The World Health Organization published in 2002 the report *Active Ageing*: *A Policy Framework* that according to the call for papers, has brought active ageing to the forefront of international public health awareness. The 2010 *Toronto Charter for Physical Activity: A Global Call for Action* was singled out in the call for papers as a key document promoting physical activity one goal of the 2002 WHO active aging policy framework. Media are to report to the public topics of importance to them. We investigated the newspaper coverage of aging well and synonymous terms such as active aging through the lens of the 2002 WHO active aging policy framework and the 2010 *Toronto Charter for Physical Activity*. As sources we used the following newspapers: *China Daily*, *The Star* (Malaysia), two UK newspapers (*The Guardian*, *The Times*), a database of 300 Canadian newspapers (*Canadian Newsstand*) and a US newspaper (*The New York Times*). The study generated data answering the following four research questions: (1) how often are the 2002 WHO active aging policy framework and the 2010 *Toronto Charter for Physical Activity* mentioned; (2) how often is the topic of active aging and terms conveying similar content (aging well, healthy aging, natural aging and successful aging) discussed; (3) which of the issues flagged as important in the 2002 WHO active aging policy framework and the 2010 *Toronto Charter for Physical Activity* are covered in the newspaper coverage of active aging and synonymous terms; (4) which social groups were mentioned in the newspapers covered. The study found a total absence of mentioning of the two key documents and a low level of coverage of “active aging” and terms conveying similar content. It found further a lack of engagement with the issues raised in the two key documents and a low level of mentioning of socially disadvantages groups. We posit that reading the newspapers we covered will not expose the reader to the two key documents and the issues linked to aging well including the need to increase physical activity.

## 1. Introduction

### 1.1. The Trend in Aging

According to the 2001 United Nations Report *World Population Ageing 1950–2050*, the number of older persons in the World will exceed the number of young persons in 2050 for the first time in history and that the reversal in relative proportions of young and old is already a reality since 1998 in the more developed regions [1]. The proportion of older persons was 8% in 1950, 10% in 2000, 11% in 2012 and is projected to reach 22% or 2 billion people by 2050 [1,2]. Furthermore within the older population, the proportion of people aged 80 years will increase from 14% in 2012 to 20% in 2050 [2]. The report *World Population Ageing 1950–2050* argues that population aging impacts “intergenerational and intragenerational equity and solidarity that are the foundations of society”, “economic growth, savings, investment and consumption, labour markets, pensions, taxation and intergenerational transfers”, “health and health care, family composition and living arrangements, housing and migration” and ”voting patterns and representation” [1]. The 2002 Madrid International Plan of Action on Ageing and the Political Declaration adopted at the Second World Assembly on Ageing (ageing and aging are two spelling versions in existence. We use aging as it gives more hits in the newspapers. We only use ageing if it is used in a title of a document as such) outlined many actions needed to deal with the increase in an aging population [3].

Aging well is increasingly discussed as a dynamic process involving use of resources, engagement within contexts and definitions of self [4]. Ageism is seen as a threat to aging well [5]. Various synonyms are used to cover aging well [6,7] such as healthy aging [8], successful aging [9,10] and natural aging. Active aging is another synonym which is used by the World Health Organization Policy Framework on Active Ageing [11] which the call for papers singles out as a very important document.

### 1.2. World Health Organization Policy Framework on Active Ageing

The World Health Organization published in 2002 the report *Active Ageing: A Policy Framework* [11] describing active aging as the “process of optimizing opportunities for health, participation and security in order to enhance quality of life as people age” [11]. The report outlines six determinants of active ageing namely (1) health and social service system; (2) behavior factors; (3) personal factors; (4) physical environment; (5) social environment and (6) economic factors (the Supplementary Material of this paper lists all the sub-measures linked to the determinants). The report identifies seven challenges for active ageing: (1) double burden of disease; (2) increase risk of disability; (3) providing care for ageing population; (4) the feminization of aging; (5) ethics and inequities; (6) the economics of an aging population and (7) the need for forging a new paradigm.

### 1.3. Active Aging Discourse beyond the WHO Report

To just give one example, the 2012 Eurobarometer (report of a survey of European citizens) on active aging, active ageing has many facets such as ability of older people to be employed, to contribution actively to society as volunteers and family carers and the ability to live independently made possible through suitable housing and infrastructure [12]. The same report notes however that the dynamic of living longer, of ageing longer is seen as a threat instead of an achievement and that older people are seen to be a burden to the working-age population [12]. The European Year for Active Ageing and Solidarity between Generations is highlighted in the report as an opportunity to encourage society to increase the opportunities for older people to partake actively in society, to change attitude and policies toward this goal and to generate best practices to achieve that goal [12].

### 1.4. The Issue of Physical Activity

Physical activity is one issue raised in need of improvement in the World Health Organization 2002 *Active Ageing: A Policy Framework* [11]. The call for papers highlights the 2010 *Toronto Charter for Physical Activity: A Global Call for Action* as a key document. The Toronto Charter outlines the utility of physical activity to improve the wellbeing, physical and mental health of people, to increase their social connectedness and quality of life [13], to generate economic benefits and to contribute to environmental sustainability [13]. However the benefits of physical activity depend on accessibility and affordability [13]. Many needed actions are highlighted in the charter such as the need for (1) adopting evidence based strategies that target the whole population as well as specific population sub groups, particularly those facing the greatest barriers; (2) embracing an equity approach aimed at reducing social and health inequalities and disparities of access to physical activity; (3) addressing the environmental, social and individual determinants of physical inactivity; (4) implementing sustainable actions in partnership at national, regional and local levels and across multiple sectors to achieve greatest impact; (5) building capacity and support training in research, practice, policy, evaluation and surveillance; (6) using a life-course approach by addressing the needs of children, families, adults and older adults; (7) advocating to decision makers and the general community for an increase in political commitment to and resources for physical activity; (8) ensuring cultural sensitivity and adapt strategies to accommodate varying “local realities”; contexts and resources and (9) facilitating healthy personal choices by making the physically active choice the easy choice. The charter promotes the implementation of a National policy and action plan and outlines numerous actions needed for it to work (for more details please read the Supplementary Material of this paper). The charter provides suggestions on how to introduce policies that support physical activities (for more details please read the Supplementary Material of this paper). The charter argues that reorienting services and funding systems toward physical activity promotions can deliver multiple benefits including better health, cleaner air, reduced traffic congestion, cost saving and greater social connectedness and gives examples of actions underway in many countries that promote physical activity (for more details please read the Supplementary Material of this paper).

### 1.5. Investigating Media Coverage

Various studies looked at portrayal of aging and the elderly in the media including in newspapers [14,15,16,17,18]. Furthermore studies looks at media coverage of various topics linked to the elderly such as elder abuse [19], successful aging [20], nursing homes [21], constructing aging [22], Alzheimer’s disease [23], attitude toward aging [24], elder in the workforce [25] and construction of the subject of care [26]. No media analysis used the WHO report or the Toronto Charter as a lens to look at active aging (and synonymous terms).

Both the 2002 WHO Policy framework for active ageing [11] and the *Toronto Charter for Physical Activity* [13] mention the importance of the media. The WHO report states:

“Older people themselves and the media must take the lead in forging a new, more positive image of ageing” [11]; “Work with groups representing older people and the media to provide realistic and positive images of active ageing, as well as educational information on active ageing. Confront negative stereotypes and ageism” [11]; “Increase awareness of the injustice of elder abuse through public information and awareness campaigns. Involve the media and young people, as well as older people in these efforts” [11]; “To advance the movement for active ageing, all stakeholders will need to clarify and popularize the term “active ageing” through dialogue, discussion and debate in the political arena, the education sector, public fora and media such as radio and television programming” [11].

The Toronto Charter in more general terms asks for action to engage the media and to use mass communication and social marketing strategies to promote increased political, community and stakeholder support for physical activity actions [13].

Wanting to involve media seems to be reasonable. *Canadian Newsstand*, a database of *n* = 300 Canadian newspapers from 1980-today has for example *n* = 20,307 articles that contain the words “aging” and “elderly” and *n* = 34,032 articles that contain the terms “aging” and “seniors” indicating an interest in the topic of aging. Diffusion of knowledge through printed media is seen for a long time as an essential part of the fabric of society to enable social participation [27,28,29] and media are seen as an influential actor in setting discussion agenda for society and in creating the boundaries within which debate takes place [29,30,31]. Media “have a vital role to play, the role of the media is essential in generating keyword recognition knowledge for the public which in turn is essential for keyword based information gathering through web-based search engines” [32] both of which are ways to increase the visibility of a topic such as active aging.

Our study adds quantitative and qualitative data to the discourse analysing the *China Daily*, *The Star* (Malaysia), two UK newspapers (*The Guardian*, *The Times*), a database of 300 Canadian newspapers (*Canadian Newsstand*) and a US newspaper (*The New York Times*). Our study answers the following research questions: (1) how often are the 2002 WHO active aging policy framework and the 2010 *Toronto Charter for Physical Activity* mentioned; (2) how often is the topic of active aging and terms conveying similar content (aging well, healthy aging, natural aging and successful aging) discussed; (3) which of the issues flagged as important in the 2002 WHO active aging policy framework and the 2010 *Toronto Charter for Physical Activity* are covered in the newspaper coverage of active aging and synonymous terms; (4) which social groups are mentioned in the newspapers covered. 

## 2. Experimental Section

### 2.1. Analytical Framework

The call for papers posits that the 2002 World Health Organization report *Active Ageing: A Policy Framework* [11] and the 2010 *Toronto Charter for Physical Activity: A Global Call for Action* [13] are important documents to be engaged with. Therefore we use these two documents as a lens to analyze the media content around active aging and synonymous terms. Section 1.2 and Section 1.4 outlined the topics we identified as issues raised by the two documents and which we used as our guidance when we read the newspaper articles.

### 2.2. Data Source

To obtain quantitative data on the research question of how often the 2002 World Health Organization report *Active Ageing: A Policy Framework* [11] and the 2010 *Toronto Charter for Physical Activity: A Global Call for Action* [13] are mentioned in newspapers we performed an deductive analysis searching various newspapers for the full and partial name of the two key documents. The following sources were investigated. We investigated *Canadian Newsstand*, a database that covers 300 Canadian newspapers from 1980-today as one focus of our study was the Toronto charter. Within this database both Canadian newspapers with national scope and reach are covered (*The Globe and Mail* and *The National Post*). As the WHO report and the Toronto Charter are envisioned to have a global impact we also investigated newspapers outside Canada. We used *The New York Times* (USA) to add one respected newspaper with national scope and reach from the USA. We also searched two UK newspapers (geographical and cultural area of Europe) with different political leaning (*The Guardian*, more liberal, and *The Times*, more conservative). We also searched two newspapers from Asia (*China Daily* and *The Star*, Malaysia) as Asia is culturally different from North America and Europe. The two Asian newspapers we chose are published in Asian countries that are different in their political system and the religious background of the country. Both are the leading English language newspapers in their respective country. This selection is not meant to be exhaustive but to give some indication as to global visibility of the documents and the visibility of aging well and the synonyms we covered.

To answer the research question of whether issues raised in the WHO report and the Toronto charter were picked up in the newspapers we first identify newspaper articles from *The New York Times*, *Calgary Herald* and *The Globe and Mail* (1980–1 May 2013) for the presence of the phrases “active aging”, “aging well”, “successful aging”, “natural aging”, “healthy aging” and “aging well”. The phrases “active aging”, “aging well”, “successful aging”, “natural aging” and “healthy aging” were found in articles of a previous literature reviews on aging well and are often seen as synonyms of aging well. One word on the spelling of the search terms; alternative spellings exist namely aging and ageing. We chose not to use the search term ageing as it gave us less hits in the newspapers then the search term aging. If we use ageing instead of aging our hits for example in the Canadian newsstand go down from 4,569 to 83 hits. Relevant articles (*n* = 158 full text articles of *The Globe and Mail*; *n* = 117 of *Calgary Herald* and *n* = 152 of *The New York Times*) were downloaded as PDF and imported into Atlas-Ti for content analysis. To generate data on which social groups are present in the newspaper coverage we used *The New York Times*, *Calgary Herald* and *The Globe and Mail* and the Canadian newsstand as sources.

To answer the research question of how often aging is used together with elderly or seniors and how often “aging well” and its synonyms are mentioned in newspapers we searched the *Canadian Newsstand* database, *Calgary Herald*, *The Globe and Mail*, *The New York Times*, *The Guardian* (UK), *The Times* (UK), *China Daily* and *The Star* (Malaysia).

### 2.3. Data Analysis

We used ATLAS.ti^©^, a qualitative data analysis software (CAQDAS) [33,34], for generating qualitative and quantitative data of the *The Globe and Mail*, *Calgary Herald* and *The New York Times* covering research question two and three.

After we imported all applicable *Globe and Mail*, *Calgary Herald* and *New York Times* articles into ATLAS.ti^©^ we performed a content analysis between 1 May 2013 and 27 August 2013 of the imported documents to identify whether any of the issues raised as important in the WHO report and the Toronto charter were dealt with in *The Globe and Mail*, *Calgary Herald* and *The New York Times* (for the results see Section 3.4 and Supplementary Material).

To answer the research question of how often the WHO report and the Toronto Charter were mentioned in various newspapers we searched all the sources on their respective websites on 21 August 2013 (result Section 3.1).

To obtain quantitative data on how often (a) aging was covered linked to elderly or seniors and (b) “aging well” and synonyms terms (active aging; healthy aging; natural aging and successful aging) were mentioned we searched *Canadian Newsstand*, *The New York Times*, *Calgary Herald*, *The Globe and Mail* through the databases accessed through the University of Calgary and *The Times*, *The Guardian*, *China Daily* and *The Star* (Malaysia) through their webpages. The search was performed on 20 October 2013) (result Section 3.2).

To obtain quantitative data on how often “physical activity” by itself and in combination with “aging/elderly” or “aging/seniors” was mentioned we searched *Canadian Newsstand* and *The New York Times*, through the databases accessed through The University of Calgary. The search was performed on 20 October 2013 (result Section 3.3).

To generate data for the research question on which social groups are mentioned in *The Globe and Mail*, *Calgary Herald* and *The New York Times* we used the Word Cruncher function of ATLAS.ti^©^ which gives one a list of words in a given document. From this list we identified social groups mentioned and the frequency they are mentioned. This work was done on 10 August 2013. We then searched also on 10 August 2013, the *Canadian Newsstand* articles that cover various aging well synonyms for these identified social groups to obtain quantitative data from *n* = 300 Canadian newspapers (result Section 3.5).

### 2.4. Limitations

We did perform an in-depth content analysis of only three North American newspapers (*Calgary Heral*d, *The Globe and Mail* and *The New York Times*). As such our findings related to the question of whether newspapers engaged with issues raised in the WHO report or the Toronto Charter do only apply to the two Canadian and the one US newspaper and the findings cannot be generalized beyond the newspapers covered. Another limitation is that our newspapers are English language based and as such we cannot account for coverage in other languages such as French coverage in the Canadian province of Quebec or any non-English language coverage in other countries. Our data can also not be used to judge other media type as we focused on newspapers. However a picture of neglect is evident in the sources we covered which we submit can be used to guide future research in investigating other media sources.

## 3. Results

### 3.1. Frequency of Mentioning of the WHO Policy Framework for Active Ageing and the Toronto Charter for Physical Activity in the Newspapers

In order to evaluate how often the WHO Policy framework for active ageing and the Toronto Charter for Physical Activity were mentioned in newspapers we searched the full text of all articles of *Canadian Newsstand* (*n* = 300 Canadian newspapers), the *China Daily* (national reach, China), *The Star* (Malaysia, national reach), *The Guardian* (UK, national reach) and *The Times* (UK, national reach) for the names of the two key documents. We did not limit this search to articles related to aging or any other prerequisite. We obtained *n* = 0 hits for the Toronto Charter for Physical Activity in all newspapers. If we looked for variation of the document title no hits were obtained either. As to the WHO Policy framework for active ageing, the exact title also had *n* = 0 hits in all newspapers covered. Variations of the name generated *n* = 3 hits in the *Canadian Newsstand* database (Policy framework on active aging, *n* = 2; Active Aging policy framework, *n* = 1). No hits were obtained in the other newspapers even with modifications to the name. The WHO document was mentioned in the three hits as follows.

“The trend got a boost more than a decade ago when the World Health Organization launched an initiative “to support communities in developing and strengthening health and social policies in an aging world”. WHO devised what it called *A Policy Framework on Active Aging*—and if you can get past the bureaucratese, it was an intriguing undertaking. The UN body solicited participation from 33 cities around the world to provide input into what they could do for their aging populations, and in 2006, Saanich was selected to participate” [35].

“The age-friendly concept was developed by the government within the Elderly Action Strategy, The strategy follows in line with the Active Aging policy framework published in 2002 by the World Health Organization (WHO), to help governments develop and combine health and social policies that take the aging population into consideration and make their environment more friendly and safe” [36].

“The World Health Organization set out a policy framework on Active Aging at the 2002 Second United Nations World Assembly on Aging. Known to researchers as the Madrid Plan, the policy framework encourages municipalities to make their communities safe and senior-friendly, promoting an active and healthy lifestyle. But Beaulieu said that the Age-Friendly Cities project includes all ages. “It is important to rethink intergenerational links”, she affirmed” [37].

The quotes of the three articles that mention the WHO document reveal a cursory engagement with the WHO document. They do not reveal the depth of the themes present in the WHO document and what it would mean for the strategies discussed in the three articles.

### 3.2. Quantitative Data on Active Aging and Related Terms (Aging Well; Healthy Aging; Natural Aging and Successful Aging)

In order to evaluate the frequency of mentioning of active aging and related terms (aging well; healthy aging; natural aging and successful aging) we searched the full text of all articles of *Canadian Newsstand* (*n* = 300 Canadian newspapers), *Calgary Herald* (Canada, local scope); T*he Globe and Mail* (Canada, national scope), *The New York Times* (USA, national scope), *China Daily* (China, national scope), *The Star* (Malaysia, national scope), *The Guardian* (UK, national scope) and *The Times* (UK, national scope) for active aging and related terms (aging well; healthy aging; natural aging and successful aging). We furthermore searched these sources for articles that covered aging and elderly and aging and seniors. 

*Canadian Newsstand* has *n* = 20,307 articles that contain the words “aging” and “elderly” and *n* = 34,032 articles that contain the terms “aging” and “seniors”. For the *Calgary Herald* the numbers are *n* = 815 articles that contain the words “aging” and “elderly” and *n* = 2,254 articles that contain the terms “aging” and “seniors”; for *The Globe and Mail* the numbers are *n* = 1,502 articles that contain the words “aging” and “elderly” and *n* = 3,063 articles that contain the terms “aging” and “seniors”; for the NYT the numbers are *n* = 4,026 articles that contain the words “aging” and “elderly” and *n* = 4,607 articles that contain the terms “aging” and “seniors”. *The Guardian* has *n* = 3,897 articles that contain the words “aging” and “elderly” and *n* = 334 articles that contain the terms “aging” and “seniors”. *The Star* (Malaysia) has *n* = 97 articles that contain the words “aging” and “elderly” and *n* = 28 articles that contain the terms “aging” and “seniors”. *China Daily* has *n* = 2,025 articles that contain the words “aging” and “elderly” and *n* = 588 articles that contain the terms “aging” and “seniors”.

These numbers indicate that the newspapers cover aging as it relates to the elderly and seniors.

However if one searches for example *The New York Times* with the term aging and seniors and “aging well” the hit number drops to *n* = 4. This number and the hit count numbers listed in Table 1 which give the overall number of articles covering “aging well” and related terms indicate that readers are exposed little to the term “aging well” as well as synonyms of it such as active aging.

**Table 1:** Frequency of occurrence of aging well related terms (active aging; aging well; healthy aging; natural aging and successful aging) in the newspaper; for *Canadian Newsstand* we also searched with the spelling ageing.

*Aging well and synonyms used for aging well*	*Canadian Newsstand (n = 300 newspapers) aging/ageing*	*Calgary Herald*	*The Globe and Mail*	*The New York Times*	*The Star (Malaysia)*	*China Daily*	*The Guardian (ageing)*	*The Times*
Active aging	684/3	21	6	8	2	0	17	16
Aging well	885/28	41	42	50	3	0	40	279 (many false positive)
Healthy aging	2,102/25	59	45	62	24	0	27	23
Natural aging	536/19	30	35	47	0	0	28	30
Successful aging	615//10	23	24	50	0	0	10	23

### 3.3. Quantitative Data on Mentioning of “Physical Activity” by Itself and in Combination with “Aging” and “Elderly” or “Seniors” and within Articles Covering Aging Well or Healthy Aging or Natural Aging or Successful Aging or Active Aging

We searched the *Canadian Newsstand* database and *The New York Times* for mentions of physical activity in articles that covered “aging“ and “elderly” or “seniors” and within articles that covered aging well or healthy aging or natural aging or successful aging or active aging.

Searching for the phrase “physical activity” by itself we found *n* = 47,634 in *Canadian Newsstand* and *n* = 1,371 in *The New York Times* indicating an interest in physical activity. Searching *Canadian Newsstand* 4,613 hits were obtained for articles covering aging well or healthy aging or natural aging or successful aging or active aging. Within these articles *n* = 362 or 7.8% of the articles covered physical activity. Searching *The New York Times* 210 hits were obtained for articles covering aging well or healthy aging or natural aging or successful aging or active aging. Within these articles *n* = 9 or 4.2% of the articles covered physical activity. These numbers indicate a low linkage of physical activity with aging well and synonyms used.

*Canadian Newsstand* has *n* = 20,307 articles that contain the words “aging” and “elderly” and *n* = 34,032 articles that contain the terms “aging” and “seniors”.

As for *Canadian Newsstand* the combination search of “physical activity” and “aging” and “elderly” generated *n* = 370 hits or 1.8% (of the hits of physical activity by itself). As for the *Canadian Newsstand* combination search of “physical activity” and “aging” and “seniors” generated *n* = 893 hits or 2.6% (of the hits of physical activity by itself). *The New York Times* has *n* = 4,024 articles on “aging” and “elderly”, *n* = 663 on “aging” and “seniors” and *n* = 1,371 on physical activity. If we search the articles on “aging” and “elderly” or “aging” and “seniors” for the term “physical activity” we obtained *n* = 43 articles in *The New York Times* for the aging/elderly articles reflecting 1.0% (of the hits of aging/elderly by itself) and *n* = 11 articles in *The New York Times* for the aging/seniors articles reflecting 1.6% (of the hits of aging/seniors by itself). This data suggest that physical activity is not much mentioned in relation to aging and the elderly/seniors in general and the low rate of coverage is not just an artifact of the aging well and synonym articles.

### 3.4. Quantitative Data of Frequency of Coverage of Issues Raised in the WHO Policy Framework for Active Ageing in the Toronto Charter for Physical Activity in the Newspapers Covered

Data so far suggest little coverage or impact that could be explicitly linked back to the WHO Policy framework for active ageing or the Toronto Charter for Physical Activity. However it could be that the issues raised in the WHO Policy framework for active ageing and in the Toronto Charter for Physical Activity are discussed in the newspapers without ever mentioning the two documents. In short we found that most themes and issues raised in the WHO Policy framework for active ageing and in the Toronto Charter for Physical Activity were not even mentioned once (see Supplementary Material for full quantitative results, Tables S1 and S2, and for full list of codes (themes and issues raised we identified in the two documents)). 

As to what was covered of the themes and issues raised by the WHO Policy framework for active ageing: in the articles covering “healthy aging” and active “aging” the topic of health promotion and disease prevention was mentioned to some extend; tobacco use was mentioned to some extend in the newspaper coverage of “successful aging” and “healthy aging”; physical activity was thematized under various aging well terms we investigated; healthy eating was covered under “healthy aging” in *Calgary Herald* and *The Globe and Mail* but not *The New York Times*; alcohol use was thematized under “healthy aging”; biology and genetics was covered under “successful aging”, “healthy aging” and natural aging; psychological factors were covered under “healthy aging”; falls were covered under various aging well term; education and literacy was covered under “healthy aging” (for full quantitative data see Table S1 in Supplementary Material). As to the issues raised in the Toronto Charter none were evident in the newspaper articles we covered (for full quantitative data see Table S2 in Supplementary Material).

### 3.5. Quantitative Data of Groups Mentioned in the Newspapers Covered

Finally both the WHO Policy framework for active ageing and the Toronto Charter for Physical Activity perceive the involvement of socially disadvantaged groups as important. We therefore investigated which social groups were mentioned how often in the newspapers.

Table 2 reveals that many socially disadvantaged groups are not at all or little mentioned (immigrant, aboriginal people, the poor, and people with disabilities). From the medical profession caregivers were less mentioned than physicians, nurses and therapists. The coverage of gerontologist differed between the newspapers with a five-fold difference between *The New York Times* and *The Globe and Mail*. Families were the group mentioned the most followed by business.

**Table 2:** Social groups mentioned in newspaper articles covering “active aging” or “natural aging” or “aging well” or “successful aging” or “healthy aging”.

Codes	*Canadian Newsstand* (*n* = 300 newspapers) 4,569 = 100%	*Calgary Herald* 161 = 100%	*The Globe and Mail* 149 = 100%	*The New York Times* 209 = 100%
Aboriginal People	0.2	0	0	0
Business	18.9	12.4	13.4	16.7
Caregiver	3.8	6.8	0.67	0.95
Corporate	1.75	3.7	2.67	4.3
Family/ies	36.3/14.7	28.5/9.9	28.1/7.38	29.6/9.5
First Nation	0.39	3.1	0	0
Gerontologist	1.51	2.4	0.67	4.78
Government	16.15	8.0	14.0	13.4
Indigenous People	0	0	0	0
Industry	6.76	8.0	8.7	8.61
Inuit	0.21	0	0	0
Immigrants	1.77	8.0	2.68	0.047
Nurses	3.91	4.3	3.35	2.87
Parents	13.59	13	8.7	11.96
People with Disability	0.91	0	0	0.047
Physicians	4.33	8.0	4.6	7.17
Society	28.4	21.1	19	20.09
Therapist	6.12	1.8	3.35	5.74
Women	28.0	29.8	36.5	36.36
The poor	0.3	0	0	1.43

## 4. Discussion

Both the WHO report [11] and the Toronto Charter [13] state that their proposed frameworks and charters will be best applied to the public through the use of media and mass communication.

We posit if mentioning of the WHO [11] and the Toronto Charter [13] by name is an indicator of success the strategies used so far failed miserably in the print media covered. However it’s less clear why they failed. Sometimes documents are covered or not based on whether they fit a certain agenda of the newspaper and its publisher [38]. However given that aging and physical activity by themselves are covered extensively in the newspapers including problems linked to aging this reason seems not to fit. Our data suggest that the terms of “active aging” and other synonyms linked to “aging well” simply are not used a lot. The lack of use of terms such as “active aging” might be another example of a simple disconnect between language used by the WHO and public health discourses and the language used in the newspapers (e.g., the phrase “social determinant of health” is one example of such disconnect [32]). The lack of coverage could also be due to a lack of clarity of aging well or active aging and that too many synonyms are used to describe aging well and that each of the synonyms has more than one meaning. Another problem of the reporting we identified is that issues raised by the two documents were rarely dealt with in the newspapers within the reporting of aging well and synonyms although many of the issues raised in the two documents were covered in the general discourse around aging. This could suggest that the newspapers use terms such as aging well and its synonyms as positive language leading to a narrative that does not fit with the discussion of problems especially problems caused by society. Rozanova looked at *The Globe and Mail* articles from 2004–2006 through the “successful aging” lens concluding that the coverage was focused on what the individual should do [20]. This lack of interest of looking at societal dynamics as problems fits with the issue that many socially disadvantaged groups were rarely mentioned in the documents covered. The low coverage of socially disadvantaged groups was also evident in the general coverage of aging as it relates to elderly and seniors and was not an artifact of the aging well coverage and was a phenomenon we encountered before for many of the newspapers covered [39].

### 4.1. Lack of Mentioning of Themes Evident and Issues Raised in the WHO Policy Framework for Active Ageing

We found that the majority of the themes and issues raised in the WHO report did not receive any coverage or were only covered once or twice in the sources we covered. The topic mentioned as action items in the WHO report are known problems mentioned within and outside of the newspapers covered. However they are not taken up in the newspaper articles that cover aging well and other synonyms such as active aging.

To give a few examples; it is for example well known that lack of mental health services are a problem for the elderly [40,41,42,43,44,45,46]. However the articles we covered did not cover mental health services that deal with elderly depression, isolation or negative mental health state.

Suicide is seen as a problem in the elderly [47,48,49]. *Canadian Newsstand* has *n* = 46,322 articles covering aging related to elderly and seniors; of which *n* = 1,098 or 2.37% cover suicide as an issue. The numbers are 2.7% for *The Globe and Mail*, 2.1% for *Calgary Herald* and 3.2% for *The New York Times*. *Calgary Herald* published recently *An epidemic of elderly suicide*; *Experts debate the causes as rates climb* [50]. *Calgary Herald*, *The Globe and Mail* and *The New York Times* cover suicide in many articles. However if we look at suicide coverage within the aging well and synonyms *Calgary Herald* had *n* = 0 articles, *The Globe and Mail* *n* = 2 articles and *The New York Times* had *n* = 4 relevant articles. They only cover the topic of suicide once within the coverage of active aging and the other aging well terms we investigated. *The New York Times* indicated that a large number of elders in America are living in depression, with high risks of suicide [51].

Violence and abuse is another well reported issue faced by the elderly and a main determinant identified by the WHO report [11] in need of improvement in order to enable active aging and aging well. Searching the newspapers for the term combination of “abuse” and “elderly” *n* = 465 hits were obtained for *Calgary Herald*; *n* = 815 for *The Globe and Mail* and *n* = 2,607 for *The New York Times*. However within articles covering active aging and related aging well terms only *n* = 6 hits were obtained with zero hits for *The New York Times*. This seems to be another failure of the newspapers covered given the following from the WHO report:

“Older people themselves and the media must take the lead in forging a new, more positive image of ageing. Political and social recognition of the contributions that older people make and the inclusion of older men and women in leadership roles will support this new image and help de-bunk negative stereotypes. Educating young people about ageing and paying careful attention to upholding the rights of older people will help to reduce and eliminate discrimination and abuse” [11].

Isolation has a huge impact on the elderly’s mental health, their physical health, their social interaction and has proven to result in illnesses and premature death [52]. As the population of elders increases, the amount of elders living in isolation also increases [52]. Isolation can be due to individuals living in poverty, or minorities being isolated due to their cultural barriers and fears [53,54]. Elders who require certain accessibility needs to get around are unable to join peers and public areas due to inaccessibility and so become isolated [55]. One article from *The Globe and Mail* states that exercise has contributed to reducing isolation in elders [56]. Another article in *Th*e *Globe and Mail* mentions the risk of isolation as one of the causes for mental decline in elders [57]. But the media we covered is not addressing causes of isolation and solutions to it.

Stereotypes are another issue mentioned in the WHO report to impact active aging in a negative way, promoting ageist behaviors [11]. In effect, elders may risk internalizing these stereotypes into their daily lives [58] which could lead to isolation and negative mental health [58]. The topic of stereotypes is not thematized in the media sources we investigated.

As a final example income is one of the economic determinants of active aging in the WHO framework. Poverty of elderly is an issue of concern investigated for some time [59,60,61]. The keyword combination search of “poverty” and “elderly” in *Calgary Herald* gained *n* = 270 hits; *n* = 882 for *The Globe and Mail* and *n* = 2,689 for *The New York Times*. Within the articles covering active aging and similar aging well terms we investigated poverty was only mentioned *n* = 17.

These examples suggest that the newspapers often cover issues mentioned in the WHO report in correlation with the elderly however they are not part of the articles that used active aging and other aging well synonyms. We posit this vastly diminishes the utility of the coverage of active aging and other aging well terms as it does not thematized the problems that have to be addressed to achieve active aging or in general aging well.

### 4.2. Frequency of Mentioning of Themes Evident and Issues Raised in the Toronto Charter for Physical Activity in the Newspapers Covered

Although the Toronto Charter for Physical Activity [13] itself was not mentioned at all it could have been that the actions it promotes were evident in the media coverage. We find that the majority of themes evident and issues raised in the Toronto Charter for Physical Activity [13] were not evident (Table S2); for example embracing an equity approach aimed at reducing physical inactivity was not covered as well coverage of social and environment issues addressing physical inactivity was absent.

As to the issue of access covered in the Toronto Charter, one article found in *The Globe and Mail* addresses the importance of elders having access to a physically active environment [62] an article in *The New York Times* addresses how accessibility to fitness centers is increasing, and elders are now able to adopt a physical active lifestyle [63]. Information on accessibility to better homes, and better public transit is also present. Accessibility to physical health is looked at by both *The New York Time* and *Calgary Herald*. A specific and detailed look at urban design to better accommodate elders is lacking though in the media articles. Rural design and rural areas are not mentioned in all three newspapers despite the known fact that rural area have numerous access issues [48]. Access issues faced by socially disadvantaged groups such as people with disabilities also aren’t mentioned. Although the newspapers promote being physically active as something that can be done by all individuals no matter their situation, it does not specify the access that is being provided to individuals with all abilities and the problems people face access-wise. In general the lack of mentioning of most of the issues raised within the Toronto charter decreases the utility of the coverage of physical activity for enticing readers to get involved in increasing physical activities among the elderly/seniors even further as the reader is not aware of the problems in existence.

### 4.3. Frequency of Mentioning of Social Groups in the Newspapers Covered

#### 4.3.1. Invisibility of Socially Disadvantaged Groups

Table 2 highlights the frequency of social groups mentioned in the newspaper articles revealing the lack of focus on socially disadvantaged groups such as indigenous people, “the poor”, ethnic minorities, immigrants, and people with disabilities. We posit it being troubling given that those social groups known to have the most problems living in Canada and the USA (disabled people, immigrants, ethnic minorities and indigenous people, the poor) [53,54,58,64,65,66,67] are hardly mentioned in the newspapers we covered.

As to disabled people the WHO report states, “The word “active” refers to continuing participation in social, economic, cultural, spiritual and civic affairs, not just the ability to be physically active or to participate in the labour force. Older people who retire from work and those who are ill or live with disabilities can remain active contributors to their families, peers, communities and nations” [11] and the Toronto Charter for Physical Activity [13] highlight the need for “Opportunities for individuals with disabilities to be physically active”. The WHO report states further that, “The Populations with low incomes, ethnic minorities and older people with disabilities are the most likely to be inactive” [11] and “It is particularly important to provide safe areas for walking and to support culturally-appropriate community activities that stimulate physical activity and are organized and led by older people themselves” [11]. Given whom the WHO report identified as being most inactive one should expect these groups to gain more attention in the media. Furthermore indigenous people and immigrants have different cultures and if support for culturally-appropriate community activities is essential [11] the public has to gain knowledge on culture of aging and aging well including active aging and the barriers faced by groups of different culture which includes immigrants, ethnic minorities and indigenous people.

Given that one action item mentioned in the Toronto Charter for Physical Activity [13] is about partnering with groups such as indigenous peoples, migrants and socially disadvantaged groups [13] the newspaper coverage does not sensitize the population to the problems these sub-populations face related to being physically active.

We posit the lack of covering immigrants and ethnic minorities, disabled people and indigenous people fits with a lack of coverage of themes of accessibility, equity and poverty.

#### 4.3.2. Health Profession

Health professionals are in large demand by society and the aging community especially with shortage of health professionals debated extensively [68,69,70]. Many of the indicators of the WHO report depend on health professionals. To give two quotes:

“Decision-makers, on governmental organizations, private industry and health and social service professionals can help foster social networks for ageing people by supporting traditional societies and community groups run by older people, voluntarism, neighbourhood helping, peer mentoring and visiting, family caregivers, intergenerational programmes and outreach services” [11]. “Changing the attitudes of health and social service providers is paramount to ensuring that their practices enable and empower individuals to remain as autonomous and independent as possible for as long as possible. Professional caregivers need to respect older people’s dignity at all times and to be careful to avoid premature interventions that may unintentionally induce the loss of independence” [11].

However the health and social service profession as well as formal and informal caregivers are hardly covered in the articles we covered and if they are, none of the challenges mentioned in the WHO report [11] are linked to them or discussed with their situation in mind. Various newspaper articles we covered advice individuals to adopt exercise, active lifestyles and engagement in order to reduce the demand of healthcare required by these elders. They also promotes physical activity and engagement as a responsibility for the elder, however they do not specifically address solution specific to healthcare providers and how they play a role in dealing with the increase needs of the elderly.

#### 4.3.3. Public Health

The call for papers states “The 2002 release by the World Health Organization of the *Active Ageing: A Policy Framework*, followed by the 2010 Global recommendations on physical activity for health have brought active ageing to the forefront of international public health awareness”. The WHO report has numerous references to public health [11]. To give one quote “Attaining the goal of active ageing will require action in a variety of sectors in addition to health and social services, including education, employment and labour, finance, social security, housing, transportation, justice and rural and urban development. While it is clear that the health sector does not have direct responsibility for policies in all of these other sectors, they belong in the broadest sense within the scope of public health because they support the goals of improved health through intersectoral action. This kind of an approach stresses the importance of the numerous different public health partners and reinforces the role of the health sector as a catalyst for action” [11].

However the public health system is only mentioned once, “The public health system can do its share by offering community health promotion and prevention programs that are designed to modify lifestyle factors like exercise and nutrition known to be associated with the incidence of frailty, poor health and disability in late life” [71] and one article mentions a public health problem, “Failure to credit the resilience and resourcefulness that successful aging requires (and that most people find within them) is a more significant public-health problem than late-life depression”[72].

If the public health field and public health interventions are that important as the WHO report suggests public health should have been more visible within the media coverage.

### 4.4. What to Do

Our study suggests that three goals need to be achieved; (a) increase in mentioning of the two documents; (b) increase of the frequency of coverage of “aging well” and synonyms such as “active aging” and (c) within this coverage of issues raised in the two documents and coverage of socially disadvantaged groups have to be increase. Question is whether one is able to raise the coverage in the newspapers. We submit that newspapers are a difficult route due to lack of influence and lack of consistent coverage. TV and Radio also are difficult and expensive for gaining constant visibility. We submit peer driven news development and topic coverage such as through Facebook which circumvents the printed media as the gatekeeper might be one way out. There are some active aging Facebook pages which we posit might be a good venue and one could make more use of social media. One might want to set up a Facebook pages for the WHO report and the Toronto Charter for increased visibility and grassroots activities. However this strategy of outreach depends on people using the internet which leaves the questions how one reaches people that do not use the internet such as many elderly people especially in low income situations. We posit that the social networking strategy has to be linked to strategies that ramp up local face to face intergenerational interactions and intensify measures that get elderly, especially elderly with disabilities, out of their isolations. As for socially disadvantaged groups the success of the strategy depends on a public that is well educated on the topic especially as it relates to socially disadvantaged groups and that the public believes that the socially disadvantaged groups should be part of it.

## 5. Conclusions and Further Research

Diffusion of knowledge through printed media is seen an essential part of the fabric of society to enable social participation [27,28,29] and policy participation of the public [32] and media are influential in setting discussion agendas and creating boundaries within which debate takes place [29,30,31]. The impact of media on the society is significant, as well as how media presents certain groups in the society [73]. Our study found that the newspapers covered failed to take up the issues seen as important in the World Health Organization 2002 Policy Framework on Active Ageing [11] and the 2010 *Toronto Charter for Physical Activity: A Global Call for Action* [13]. As such the newspapers covered were not creating a discussion agenda for society as envisioned by the two documents. They lacked coverage of many stakeholders who are identified in the literature as facing problems with aging well such as disabled people, immigrants, “the poor” “indigenous people” and other socially disadvantaged groups.

The newspapers covered did not sensitize the reader as to the challenges identified by the World Health organization 2002 Policy Framework on Active Ageing [11] and the 2010 *Toronto Charter for Physical Activity: A Global Call for Action* [13]. Given the lack of control over newspaper coverage it might be more useful to focus on social media linked to local feet on the ground.

Our work focused mostly on Canadian newspapers and one US newspaper with only two of the four research questions investigating newspapers beyond North American (UK, China, and Malaysia). Future research could look at other media sources to generate empirical data on the other research questions we tackled such as whether newspapers from other cultures covered more the issues raised by the WHO report [11] and the Toronto Charter for physical activity [13] and which social groups they cover.

## Supplementary Files

Supplementary File 1 Supplementary Information (PDF, 190 KB)
