# Supplementary material for: Analysis of Newspaper Coverage of Active Aging through the Lens of the 2002 World Health Organization Active Ageing Report: A Policy Framework and the 2010 Toronto Charter for Physical Activity: A Global Call for Action
Source: Int J Environ Res Public Health. 2013 Dec 5;10(12):6799–819. doi: 10.3390/ijerph10126799 (PMC3881142; doi:10.3390/ijerph10126799)
Supplement: Supplementary File 1 — Supplementary Information (PDF, 190 KB) [file ijerph-10-06799-s001.pdf]

Supplementary Information

Analysis of Newspaper Coverage of Active Aging through the Lens of the 2002 World Health Organization Report Active Ageing: A Policy Framework and the 2010 Toronto Charter for Physical Activity: A Global Call for Action

---

1. Method

The below listed issues and themes identified in the World Health Organization policy framework on active ageing [11] *2010 Toronto Charter for Physical Activity: A Global Call for Action* [13] were used as codes. We read the newspaper articles with these codes in mind ascertaining the frequency of how often one of the codes were present (for full method please refer back to the main paper).

1.1. Issues Raised and Themes Identified in the World Health Organization Policy Framework on Active Ageing

The report outlined six determinants of active ageing each with various sub-measures. One determinant covers Health and social service system and has the following sub-measures: Health promotion and disease prevention, curative services and long-term care. The second determinant is linked to behavior covering mental health services, tobacco use, physical activity, healthy eating, oral health, alcohol use, iatrogenesis, medication and adherence. The third determinant covers personal factors such as biology and genetics, psychological factors. The fourth determinant covers the physical environment such as safe housing, falls, clean water, clean air and safe food. The fifth determinant covers social environment covering social support, violence and abuse, education and literacy and social protection. The sixth determinant covers economic factors such as income and work (employment). The report furthermore outlined seven challenges for active ageing: double burden of disease, increase risk of disability, providing care for ageing population, the feminization of aging, ethics and inequities, the economics of an aging population and the need for forging a new paradigm.

1.2. Issues Raised and Themes Identified in the 2010 Toronto Charter for Physical Activity: A Global Call for Action

The following themes are evident in the charter: (1) Adopt evidence based strategies that target the whole population as well as specific population sub groups, particularly those facing the greatest barriers; (2) Embrace an equity approach aimed at reducing social and health inequalities and disparities of access to physical activity; (3) Address the environmental, social and individual determinants of physical inactivity; (4) Implement sustainable actions in partnership at national, regional and local levels and across multiple sectors to achieve greatest impact; (5) Build capacity and support training in research, practice, policy, evaluation and surveillance; (6) Use a life-course approach by addressing the needs of children, families, adults and older adults; (7) Advocate to

decision makers and the general community for an increase in political commitment to and resources for physical activity; (8) Ensure cultural sensitivity and adapt strategies to accommodate varying "local realities"; contexts and resources and (9) Facilitate healthy personal choices by making the physically active choice the easy choice.

As for the implementation of a National policy and action plan which the Charter sees as an action item the following was seen as needed: (1) Gain input from a broad constituency of relevant stakeholders; (2) Identify clear leadership for physical activity, which may come from any government sector, other relevant non- government agencies or from a cross sector collaboration; (3) Describe the roles and actions that government, not-for-profit, volunteer and private sector organisations at national, regional and local levels should take to implement the plan and promote physical activity; (4) Provide an implementation plan that defines accountability, timelines and funding; (5) Include combinations of different strategies to influence individual, social, cultural and built environment factors that will inform, motivate and support individuals and communities to be active, in ways that are safe and enjoyable and (6) Adopt evidence based guidelines on physical activity and health.

As to how to introduce policies that support physical activities another action item promoted by the Charter, the Charter mentioned the need for (1) clear national policy with objectives for increasing physical activity that state by how much and by when. All sectors can share common goal(s) and identify their contribution; (2) Urban and rural planning policies and design guidelines that support walking, cycling, public transport, sport and recreation with a particular focus on equitable access and safety; (3) Fiscal policies such as subsidies, incentives and tax deductions that may support participation in physical activity or taxation to reduce obstacles. For example, tax incentives on physical activity equipment or club membership; (4) Workplace policies that support infrastructure and programs for physical activity and promote active transport to and from work; process of developing this Charter which we hope will provide a clear call for action, to health and non health sectors, and be applicable and relevant to all countries but particularly low and middle income countries; (5) Education policies that support high quality compulsory physical education, active travel to school, physical activity during the school day and healthy school environments; (6) Sport and recreation policy and funding systems that prioritise increased community participation by all members of the community; (7) Advocacy to engage the media to promote increased political commitment to physical activity. For example, "Report Cards" or civil society reports on the implementation of physical activity action to increase accountability; (8) Mass communication and social marketing campaigns to increase community and stakeholder support for physical activity action.

The charter also asks for the reorientation of services and funding to prioritise physical activity. The charter states that action will require a reorientation of priorities in favour of health enhancing physical activity. The charter argues that reorienting services and funding systems can deliver multiple benefits including better health, cleaner air, reduced traffic congestion, cost saving and greater social connectedness.

The charter gives example of actions underway in many countries such as under the section education the charter mentions (1) Education systems that prioritise high-quality compulsory physical education curriculum with an emphasis on non- competitive sports in schools and enhancing physical education training for all teachers; (2) Physical activity programs that focus on a range of activities that maximise participation regardless of skill level and that focus on enjoyment; (3) Opportunity for

students to be active during class, in breaks, at lunch time and after school; (4) Transport policies and services, that prioritise and fund, walking, cycling and public transit infrastructure; (5) Building codes that encourage or support physical activity; (6) Trails in national parks and preserved areas to increase access.

As to planning and environment the charter mentions the following examples: (1) Evidence based urban design that support walking, cycling and recreational physical activity and (2) Urban design that provides opportunities for sport, recreation and physical activity by increasing access to public space where people of all ages and abilities can be physically active in urban and rural settings.

As to the workplace the charter highlights: (1) Workplace programs that encourage and support employees and their families to lead active lifestyles; (2) Facilities that encourage participation in physical activity and (3) Incentives for active commuting to work or by public transport rather than by car.

As for the examples for the section sport, parks and recreation the charter highlights: (1) Mass participation and sports for all, including those least likely to participate; (2) Infrastructure for recreational activities across the life-course; (3) Opportunities for individuals with disabilities to be physically active; (4) Building capacity among those who deliver sport through increased training on physical activity.

As to health the charter mentions: (1) Greater priority and resourcing of prevention and health promotion including physical activity; (2) Screening of patients/clients for levels of physical activity at every primary care consultation, and provision of brief, structured counselling and referral to community programs for insufficiently active patients; (3) For patients with diseases/conditions such as diabetes, cardiovascular disease, some cancers or arthritis, screening by health and exercise professionals for contraindications and advice on physical activity as part of treatment, management and review plans.

2. Results

A summary of the results of Tables S1 and S2 can be found in the main paper. The below highlights the quantitative results in detail.

Table S1. Coverage of themes evident and issues raised in the WHO Policy framework for active ageing in the newspapers covered.

	**Active Aging**	**Successful Aging**	**Mindful Aging**	**Healthy Aging**	**Aging Well**	**Longitudinal Aging**	**Advanced Aging**	**Natural Aging**
The Determinants of Active Ageing: Determinants Related to Health and social service system in each newspaper								
Health Promotion and Disease Prevention								
Calgary Herald	4	1	0	28	2	0	0	0
The Globe and Mail	3	1	0	14	7	0	0	1
The New York Times	2	1	0	0	1	0	0	0

Table S1. *Cont.*

	Active Aging	Successful Aging	Mindful Aging	Healthy Aging	Aging Well	Longitudinal Aging	Advanced Aging	Natural Aging
				Curative Services				
Calgary Herald	0	0	0	0	0	0	0	0
The Globe and Mail	0	0	0	0	0	0	0	0
The New York Times	0	0	0	0	0	0	0	0
Long Term Care								
Calgary Herald	0	0	0	1	0	0	0	0
The Globe and Mail	0	0	0	0	0	0	0	0
The New York Times	0	0	0	0	0	0	0	0
				**Behavioural Determinants**				
				Mental Health Services				
Calgary Herald	0	0	0	0	0	0	0	0
The Globe and Mail	1	2	0	1	1	0	0	0
The New York Times	1	0	0	0	0	0	1	0
				Tobacco Use				
Calgary Herald	0	1	0	4	1	0	0	0
The Globe and Mail	0	4	0	7	2	0	0	1
The New York Times	0	1	0	0	4	0	0	1
				Physical Activity				
Calgary Herald	10	9	1	17	3	1	0	0
The Globe and Mail	6	8	0	18	8	0	0	4
The New York Times	7	3	0	0	5	0	0	4
				Healthy Eating				
Calgary Herald	0	2	0	18	5	0	0	0
The Globe and Mail	0	2	0	23	4	0	0	1
The New York Times	0	2	0	0	5	0	0	5

Table S1. *Cont.*

	Active Aging	Successful Aging	Mindful Aging	Healthy Aging	Aging Well	Longitudinal Aging	Advanced Aging	Natural Aging
				Oral Health				
Calgary Herald	1	1	0	0	0	0	0	0
The Globe and Mail	0	0	0	1	1	0	0	1
The New York Times	0	0	0	0	0	0	0	0
				Alcohol				
Calgary Herald	1	5	0	9	3	0	0	0
The Globe and Mail	0	0	0	15	3	0	0	1
The New York Times	0	2	0	0	5	0	0	2
				Iatrogenesis				
Calgary Herald	0	0	0	0	0	0	0	0
The Globe and Mail	0	0	0	0	0	0	0	1
The New York Times	0	0	0	0	0	0	0	0
				Medications				
Calgary Herald	0	1	0	0	0	0	0	0
The Globe and Mail	0	2	0	4	1	0	0	3
The New York Times	0	3	0	0	2	0	0	1
Adherence								
Calgary Herald	0	0	0	0	0	0	0	0
The Globe and Mail	0	0	0	0	1	0	0	0
The New York Times`	0	0	0	1	0	0	0	0
				**Determinants Related to Personal Factors**				
				Biology and Genetics				
Calgary Herald	1	10	1	10	5	0	0	0
The Globe and Mail	0	2	0	20	0	0	0	6
The New York Times	0	2	0	0	7	0	0	14

Table S1. *Cont.*

	Active Aging	Successful Aging	Mindful Aging	Healthy Aging	Aging Well	Longitudinal Aging	Advanced Aging	Natural Aging
Psychological Factors								
Calgary Herald	0	0	0	6	2	1	0	0
The Globe and Mail	0	5	0	13	2	0	0	1
The New York Times	1	2	0	0	0	0	0	0
**Determinants Related to the Physical Environment**								
Safe Housing								
Calgary Herald	1	0	0	0	0	0	0	0
The Globe and Mail	1	1	0	0	0	0	0	0
The New York Times	0	0	0	0	0	0	0	0
Falls								
Calgary Herald	1	7	0	13	0	0	0	0
The Globe and Mail	2	0	0	6	1	0	0	0
The New York Times	6	4	0	0	1	0	0	3
Clean Water, Clean Air and Safe Foods								
Calgary Herald	0	1	0	0	0	0	0	0
The Globe and Mail	0	0	0	2	0	0	0	0
The New York Times	0	0	0	0	0	0	0	0
**Determinants Related to the Social Environment**								
Social Support								
Calgary Herald	0	0	1	5	1	0	0	0
The Globe and Mail	2	1	0	0	0	0	0	0
The New York Times	0	1	0	0	0	0	0	0
Violence and Abuse								
Calgary Herald	0	0	0	2	0	0	0	0
The Globe and Mail	2	0	0	2	0	0	0	0
The New York Times	0	0	0	0	0	0	0	0

Table S1. *Cont.*

	Active Aging	Successful Aging	Mindful Aging	Healthy Aging	Aging Well	Longitudinal Aging	Advanced Aging	Natural Aging
				Education and Literacy				
Calgary Herald	1	2	0	8	1	0	0	0
The Globe and Mail	1	4	0	7	2	0	0	2
The New York Times	1	2	0	0	3	0	0	1
				Social Protection				
Calgary Herald	0	0	0	0	0	0	0	0
The Globe and Mail	0	0	0	0	1	0	0	0
The New York Times	0	0	0	0	0	0	0	0
				**Economic Determinants**				
				Income				
Calgary Herald	0	3	0	2	0	0	0	0
The Globe and Mail	1	4	0	4	2	0	0	1
The New York Times	0	0	0	0	0	0	0	0
				Work				
Calgary Herald	1	0	0	2	4	0	0	0
The Globe and Mail	0	5	0	3	2	0	0	2
The New York Times	0	3	0	0	4	0	0	0
				**Challenge 1: The Double Burden of Disease**				
Calgary Herald	0	0	0	0	0	0	0	0
The Globe and Mail	0	1	0	2	0	0	0	0
The New York Times	0	0	0	0	0	0	0	1
				**Challenge 2: Increased Risk of Disability**				
Calgary Herald	0	0	0	1	0	0	0	0
The Globe and Mail	0	0	0	1	0	0	0	0
The New York Times	0	1	0	0	3	0	0	0

Table S1. *Cont.*

	Active Aging	Successful Aging	Mindful Aging	Healthy Aging	Aging Well	Longitudinal Aging	Advanced Aging	Natural Aging
**Challenge 3: Providing Care for Ageing Populations**								
Calgary Herald	0	0	0	7	0	0	0	0
The Globe and Mail	0	1	0	2	0	0	0	0
The New York Times	1	0	0	0	0	0	0	0
**Challenge 4: The Feminization of Ageing**								
Calgary Herald	0	0	0	2	1	0	0	0
The Globe and Mail	1	5	0	3	6	0	0	2
The New York Times	0	5	0	0	4	0	0	1
**Challenge 5: Ethics and Inequities**								
Calgary Herald	0	1	0	1	0	0	0	0
The Globe and Mail	0	0	0	2	0	0	0	0
The New York Times	0	0	0	0	0	0	0	1
**Challenge 6: The Economics of an Ageing Population**								
Calgary Herald	1	1	0	2	1	0	0	0
The Globe and Mail	0	2	0	2	1	0	0	1
The New York Times	1	2	0	0	5	0	0	1
**Challenge 7: Forging a New Paradigm**								
Calgary Herald	1	0	0	0	0	0	0	0
The Globe and Mail	0	0	0	0	0	0	0	0
The New York Times	0	0	0	0	0	0	0	0

Table S2. Coverage of themes evident and issues raised in the Toronto Charter for Physical Activity.

	Active Aging	Successful Aging	Advanced Aging	Aging Well	Healthy Aging	Longitudinal Aging	Mindful Aging	Natural Aging
1. Adopt evidence based strategies that target the whole population as well as specific population sub groups, particularly those facing the greatest barriers;								
Calgary Herald	0	0	0	0	1	0	0	0
The Globe and Mail	0	1	0	0	3	0	0	0
The New York Times	0	0	0	0	0	0	0	0

Table S2. *Cont.*

	Active aging	Successful Aging	Advanced Aging	Aging Well	Healthy Aging	Longitudinal Aging	Mindful Aging	Natural Aging
2. Embrace an equity approach aimed at reducing social and health inequalities and disparities of access to physical activity;								
Calgary Herald	0	0	0	0	0	0	0	0
The Globe and Mail	0	0	0	0	0	0	0	0
The New York Times	0	0	0	0	0	0	0	0
3. Address the environmental, social and individual determinants of physical inactivity;								
Calgary Herald	1	0	0	0	0	0	0	0
The Globe and Mail	0	2	0	0	0	0	0	0
The New York Times	1	0	0	0	0	0	0	0
4. Implement sustainable actions in partnership at national, regional and local levels and across multiple sectors to achieve greatest impact;								
Calgary Herald	4	0	0	0	3	0	0	0
The Globe and Mail	1	1	0	0	0	0	0	0
The New York Times	2	1	0	1	0	0	0	2
5. Build capacity and support training in research, practice, policy, evaluation and surveillance;								
Calgary Herald	0	0	0	0	0	0	0	0
The Globe and Mail	0	0	0	0	0	0	0	0
The New York Times	0	0	0	0	0	0	0	0
6. Use a life-course approach by addressing the needs of children, families, adults and older adults;								
Calgary Herald	0	0	0	0	3	0	0	0
The Globe and Mail	1	1	0	0	1	0	0	1
The New York Times	0	0	0	1	0	0	0	0
7. Advocate to decision makers and the general community for an increase in political commitment to and resources for physical activity;								
Calgary Herald	0	0	0	0	0	0	0	0
The Globe and Mail	0	0	0	0	0	0	0	0
The New York Times	0	0	0	0	0	0	0	0

Table S2. *Cont.*

	Active Aging	Successful Aging	Advanced Aging	Aging Well	Healthy Aging	Longitudinal Aging	Mindful Aging	Natural Aging
8. Ensure cultural sensitivity and adapt strategies to accommodate varying "local realities", contexts and resources;								
Calgary Herald	0	0	0	0	0	0	0	0
The Globe and Mail	0	0	0	0	0	0	0	0
The New York Times	0	0	0	0	0	0	0	0
9. Facilitate healthy personal choices by making the physically active choice the easy choice.								
Calgary Herald	0	0	0	0	0	0	0	0
The Globe and Mail	0	0	0	0	0	0	0	0
The New York Times	0	0	0	0	0	0	0	0
1. Implement a national policy and action plan								
• Gain input from a broad constituency of relevant stakeholders;								
Calgary Herald	0	0	0	0	1	0	0	0
The Globe and Mail	0	0	0	0	0	0	0	0
The New York Times	0	0	0	0	0	0	0	0
• Identify clear leadership for physical activity, which may come from any government sector, other relevant non-government agencies or from a cross sector collaboration;								
Calgary Herald	2	0	0	0	0	0	0	0
The Globe and Mail	0	0	0	0	0	0	0	0
The New York Times	0	0	0	0	0	0	0	0
• Describe the roles and actions that government, not-for-profit, volunteer and private sector organisations at national, regional and local levels should take to implement the plan and promote physical activity;								
Calgary Herald	2	0	0	0	0	0	0	0
The Globe and Mail	0	0	0	0	0	0	0	0
The New York Times	0	0	0	0	0	0	0	0
• Provide an implementation plan that defines accountability, timelines and funding;								
Calgary Herald	0	0	0	0	0	0	0	0
The Globe and Mail	0	0	0	0	0	0	0	0
The New York Times	0	0	0	0	0	0	0	0

Table S2. *Cont.*

	Active Aging	Successful Aging	Advanced Aging	Aging Well	Healthy Aging	Longitudinal Aging	Mindful Aging	Natural Aging
• Include combinations of different strategies to influence individual, social, cultural and built environment factors that will inform, motivate and support individuals and communities to be active, in ways that are safe and enjoyable;								
Calgary Herald	0	0	0	0	0	0	0	0
The Globe and Mail	0	0	0	0	1	0	0	0
The New York Times	0	0	0	0	0	0	0	0
• Adopt evidence based guidelines on physical activity and health.								
Calgary Herald	0	0	0	0	1	1	0	0
The Globe and Mail	0	1	0	0	1	0	0	1
The New York Times	1	2	0	0	0	0	0	0
2. Introduce policies that support physical activity								
• Clear national policy with objectives for increasing physical activity that state by how much and by when. All sectors can share common goal(s) and identify their contribution;								
Calgary Herald	0	0	0	0	0	0	0	0
The Globe and Mail	0	0	0	0	0	0	0	0
The New York Times	0	0	0	0	0	0	0	0
• Urban and rural planning policies and design guidelines that support walking, cycling, public transport, sport and recreation with a particular focus on equitable access and safety;								
Calgary Herald	0	0	0	0	1	0	0	0
The Globe and Mail	0	0	0	0	0	0	0	0
The New York Times	0	0	0	0	0	0	0	0
• Fiscal policies such as subsidies, incentives and tax deductions that may support participation in physical activity or taxation to reduce obstacles. For example, tax incentives on physical activity equipment or club membership;								
Calgary Herald	0	0	0	0	0	0	0	0
The Globe and Mail	0	0	0	0	0	0	0	0
The New York Times	0	0	0	0	0	0	0	0

Table S2. *Cont.*

	Active Aging	Successful Aging	Advanced Aging	Aging Well	Healthy Aging	Longitudinal Aging	Mindful Aging	Natural Aging
• Workplace policies that support infrastructure and programs for physical activity and promote active transport to and from work; process of developing this Charter which we hope will provide a clear call for action, to health and non health sectors, and be applicable and relevant to all countries but particularly low and middle income countries.								
Calgary Herald	0	0	0	0	0	0	0	0
The Globe and Mail	0	0	0	0	0	0	0	0
The New York Times	0	0	0	0	0	0	0	0
• Education policies that support high quality compulsory physical education, active travel to school, physical activity during the school day and healthy school environments;								
Calgary Herald	0	0	0	0	0	0	0	0
The Globe and Mail	0	0	0	0	0	0	0	0
The New York Times	0	0	0	0	0	0	0	0
• Sport and recreation policy and funding systems that prioritise increased community participation by all members of the community;								
Calgary Herald	0	0	0	0	0	0	0	0
The Globe and Mail	0	0	0	0	0	0	0	0
The New York Times	0	0	0	0	0	0	0	0
• Advocacy to engage the media to promote increased political commitment to physical activity. For example, "Report Cards" or civil society reports on the implementation of physical activity action to increase accountability;								
Calgary Herald	0	0	0	0	0	0	0	0
The Globe and Mail	0	0	0	0	0	0	0	0
The New York Times	0	0	0	0	0	0	0	0
• Mass communication and social marketing campaigns to increase community and stakeholder support for physical activity action.								
Calgary Herald	0	0	0	0	0	0	0	0
The Globe and Mail	0	0	0	0	0	0	0	0
The New York Times	0	0	0	0	0	0	0	0

Table S2. *Cont.*

	Active Aging	Successful Aging	Advanced Aging	Aging Well	Healthy Aging	Longitudinal Aging	Mindful Aging	Natural Aging
3. Reorient services and funding to prioritise physical activity								
In most countries, successful action to promote physical activity will require a reorientation of priorities in favour of health enhancing physical activity. Reorienting services and funding systems can deliver multiple benefits including better health, cleaner air, reduced traffic congestion, cost saving and greater social connectedness. Examples of actions underway in many countries include:								
Calgary Herald	0	0	0	0	0	0	0	0
The Globe and Mail	0	0	0	0	0	0	0	0
The New York Times	0	0	0	0	0	0	0	0
In education:	0	0	0	0	0	0	0	0
• Education systems that prioritise high-quality compulsory physical education curriculum with an emphasis on non-competitive sports in schools and enhancing physical education training for all teachers;								
Calgary Herald	0	0	0	0	0	0	0	0
The Globe and Mail	0	0	0	0	0	0	0	0
The New York Times	0	0	0	0	0	0	0	0
• Physical activity programs that focus on a range of activities that maximise participation regardless of skill level and that focus on enjoyment;								
Calgary Herald	3	0	0	0	2	0	0	0
The Globe and Mail	1	0	0	0	0	0	0	0
The New York Times	1	0	0	1	0	0	0	0
• Opportunity for students to be active during class, in breaks, at lunch time and after school.								
Calgary Herald	0	0	0	0	0	0	0	0
The Globe and Mail	0	0	0	0	0	0	0	0
The New York Times	0	0	0	0	0	0	0	0
In transportation and planning:	0	0	0	0	0	0	0	0
• Transport policies and services, that prioritise and fund, walking, cycling and public transit infrastructure;								
Calgary Herald	0	0	0	0	0	0	0	0
The Globe and Mail	0	0	0	0	0	0	0	0
The New York Times	0	0	0	0	0	0	0	0

Table S2. *Cont.*

	Active Aging	Successful Aging	Advanced Aging	Aging Well	Healthy Aging	Longitudinal Aging	Mindful Aging	Natural Aging
• Building codes that encourage or support physical activity:								
Calgary Herald	0	0	0	0	0	0	0	0
The Globe and Mail	0	0	0	0	0	0	0	0
The New York Times	0	0	0	0	0	0	0	0
• Trails in national parks and preserved areas to increase access.								
Calgary Herald	0	0	0	0	0	0	0	0
The Globe and Mail	0	0	0	0	0	0	0	0
The New York Times	0	0	0	0	0	0	0	0
In planning and environment:								
• Evidence based urban design that support walking, cycling and recreational physical activity;								
Calgary Herald	1	0	0	0	0	0	0	0
The Globe and Mail	0	0	0	0	0	0	0	0
The New York Times	0	0	0	0	0	0	0	0
• Urban design that provides opportunities for sport, recreation and physical activity by increasing access to public space where people of all ages and abilities can be physically active in urban and rural settings.								
Calgary Herald	0	0	0	0	0	0	0	0
The Globe and Mail	0	0	0	0	0	0	0	0
The New York Times	0	0	0	0	0	0	0	0
In workplace:								
• Workplace programs that encourage and support employees and their families to lead active lifestyles;								
Calgary Herald	0	0	0	0	0	0	0	0
The Globe and Mail	0	0	0	0	0	0	0	0
The New York Times	0	0	0	0	0	0	0	0
• Facilities that encourage participation in physical activity;								
Calgary Herald	2	0	0	0	9	0	0	0
The Globe and Mail	2	0	0	2	0	0	0	0
The New York Times	6	0	1	0	0	0	0	0

Table S2. *Cont.*

	Active Aging	Successful Aging	Advanced Aging	Aging Well	Healthy Aging	Longitudinal Aging	Mindful Aging	Natural Aging
• Incentives for active commuting to work or by public transport rather than by car.								
Calgary Herald	0	0	0	0	0	0	0	0
The Globe and Mail	0	0	0	0	0	0	0	0
The New York Times	0	0	0	0	0	0	0	0
In sport, parks and recreation:								
• Mass participation and sports for all, including those least likely to participate;								
Calgary Herald	0	0	0	0	0	0	0	0
The Globe and Mail	0	0	0	0	0	0	0	0
The New York Times	0	0	0	0	0	0	0	0
• Infrastructure for recreational activities across the life-course;								
Calgary Herald	0	0	0	0	0	0	0	0
The Globe and Mail	0	0	0	0	0	0	0	0
The New York Times	0	0	0	0	0	0	0	0
• Opportunities for individuals with disabilities to be physically active;								
Calgary Herald	0	0	0	0	0	0	0	0
The Globe and Mail	1	0	0	0	0	0	0	0
The New York Times	0	0	0	0	0	0	0	0
• Building capacity among those who deliver sport through increased training on physical activity.								
Calgary Herald	0	0	0	0	0	0	0	0
The Globe and Mail	0	0	0	0	0	0	0	0
The New York Times	0	0	0	0	0	0	0	0
In health:								
• Greater priority and resourcing of prevention and health promotion including physical activity								
Calgary Herald	1	0	0	0	0	0	0	0
The Globe and Mail	0	0	0	0	0	0	0	0
The New York Times	0	0	0	1	0	0	0	0

Table S2. *Cont.*

	Active Aging	Successful Aging	Advanced Aging	Aging Well	Healthy Aging	Longitudinal Aging	Mindful Aging	Natural Aging
• Screening of patients/clients for levels of physical activity at every primary care consultation, and provision of brief, structured counselling and referral to community programs for insufficiently active patients;								
Calgary Herald	0	0	0	0	0	0	0	0
The Globe and Mail	0	0	0	0	0	0	0	0
The New York Times	0	0	0	0	0	0	0	0
• For patients with diseases/conditions such as diabetes, cardiovascular disease, some cancers or arthritis, screening by health and exercise professionals for contraindications and advice on physical activity as part of treatment, management and review plans.								
Calgary Herald	0	0	0	0	2	0	0	0
The Globe and Mail	0	0	0	0	0	0	0	0
The New York Times	0	0	0	0	0	0	0	0

© 2013 by the authors; licensee MDPI, Basel, Switzerland. This article is an open access article distributed under the terms and conditions of the Creative Commons Attribution license (http://creativecommons.org/licenses/by/3.0/).
